# Risk factors associated with carbapenem-resistant Gram-negative bacterial infections in intensive care units in Jordan

**DOI:** 10.1186/s12879-026-13526-w

**Published:** 2026-09-26

**Authors:** Mohammad Gharaibeh, Omar Sayyouh, Mona El-Shokry, Salwa Nasrat, Mohamed Ramadan, Wiam Khraisat, Lina Alamer, Alia Natour, Mohammad Aljbour, Zainab Abushawer, Najla’a Abdallah, Nisreen Migdadi, Suzan Harb, Essam Awad, Enas Bataienh, Suzanne Battah, Ruba Ikermawi, Mayar Said, Mohammad Alhawarat, Tamer Osman

**Affiliations:** 1 Ministry of Health, Amman, Jordan; 2 Goldbelt Apex, Contract Support to US Naval Medical Research Unit EURAFCENT, Cairo, Egypt; 3 Ain Shams University, Cairo, Egypt; 4 US Naval Medical Research Unit EURAFCENT, Cairo, Egypt

**Keywords:** Carbapenem resistance, Gram-negative, Risk factors, Antimicrobial resistance, Healthcare-associated infections, Intensive care units

## Abstract

**Background:**

Carbapenem-resistant Gram-negative bacteria (CR-GNB) pose major challenges in intensive care units (ICUs) due to limited treatment options and high mortality. Data describing their predictors in Jordan are scarce. This study aimed to identify risk factors associated with CR-GNB infections among ICU patients.

**Methodology:**

A retrospective analysis was conducted using national surveillance data from 18 ICUs across 13 public hospitals in Jordan between September 2018 and September 2024. Patients with confirmed Gram-negative infections were included. Laboratory testing followed CLSI M100 standards. Logistic regression was performed to determine independent predictors of carbapenem resistance.

**Results:**

Of 1,180 Gram-negative isolates tested, 451 (38%) were carbapenem-resistant. *Acinetobacter baumannii* and *Klebsiella* spp. were the predominant resistant pathogens, while *E.coli* was mainly carbapenem-susceptible. Independent predictors of carbapenem resistance identified by multivariate logistic regression were: ICU-acquired infection (OR: 2.44; 95% CI: 1.31–4.54; *p* = 0.004), pneumonia (OR: 2.77; 95% CI: 1.48–5.23; *p* = 0.001), invasive device use (OR: 3.37; 95% CI: 1.28–9.95; *p* = 0.019), and admission to high-risk ICU units (OR: 3.33; 95% CI: 2.06–5.48; *p* < 0.001). Mortality was significantly higher among CR-GNB patients (69.8%) compared to carbapenem-susceptible patients (42.0%), with a 3.15-fold increased risk of death (OR: 3.149; 95% CI: 2.392–4.145; *p* < 0.001).

**Conclusion:**

The findings of this study underscore the need for strategies to prevent and control carbapenem-resistant infections, particularly in high-risk populations such as patients with invasive devices and those diagnosed with pneumonia. Enhanced infection control measures, including strict hand hygiene practices, judicious antibiotic stewardship, and proper management of invasive devices, are essential to minimize the transmission of carbapenem-resistant bacteria in healthcare settings.

## Introduction

Gram-negative bacteria are a major component of hospital environments worldwide, accounting for approximately 49% to 62% of pathogens isolated from intensive care unit (ICU) patients with infections [1, 2]. Their growing prevalence, coupled with the increasing burden of antimicrobial resistance, has made them a central focus of studies examining hospital microbial ecosystems [3]. Multidrug resistance among Gram-negative pathogens, particularly *E.coli*, *Klebsiella pneumoniae*, *Enterobacter spp.*,* Acinetobacter baumannii*, and *Pseudomonas aeruginosa*, including extended-spectrum beta-lactamase-producing Enterobacterales (ESBL) and carbapenem-resistant Enterobacterales (CRE), has been reported worldwide, with limited therapeutic options. Carbapenems remain highly effective against many bacterial species and are less affected by most beta-lactam resistance mechanisms, making them essential agents for the treatment of multidrug-resistant infections [4]. They are also generally safer and associated with fewer adverse effects than other last-line agents, such as colistin [5]. Nevertheless, carbapenem resistance among clinical Gram-negative isolates, particularly in ICUs, has increased steadily since 2005 [6]. Reflecting the growing global concern, the World Health Organization (WHO) placed carbapenems in the “Reserve” category of its Access, Watch, Reserve (AWaRe) classification of antimicrobials [7]. More recently, in 2024, the WHO classified carbapenem-resistant Enterobacterales and Acinetobacter baumannii as critical priority pathogens, while carbapenem-resistant *Pseudomonas aeruginosa* was designated a high-priority threat to public health [8].

ICUs have been recognized as an ideal environment for carbapenem-resistant Gram-negative pathogens, enabled by the immunocompromised status of patients, prolonged antimicrobial treatments, and associated co-morbidities [9]. In low and middle-income countries (LMICs), ICU-acquired infection rates (22.4%) are higher than those observed in ICUs in Western Europe (9.3–19.5%) [2, 3], . Several publications have emerged from the Middle East, reporting the alarming rise of carbapenem resistance in hospital settings [6, 10, 11]. The transfer of resistance genes through mobile genetic elements among diverse bacterial species is a key driver of carbapenem resistance [12]. In Jordan, several studies have confirmed the prevalence of carbapenem-resistant Gram-negative bacilli, with bla _*NDM*_ and bla_*OXA−48*_ identified as the most common resistance genes in hospitals [13, 14]. However, the factors associated with the spread of carbapenem resistance remain insufficiently understood and require further investigation to guide infection prevention strategies and antibiotic stewardship policies. Early identification of patients at high risk for carbapenem-resistant infections may support targeted clinical management and help inform evidence-based interventions [15].

The aim of this study was to identify the risk factors associated with carbapenem resistance in ICUs among patients infected with Gram-negative pathogens.

To our knowledge, this is the first study to address the risk factors associated with the spread of carbapenem resistance in ICUs in Jordan.

## Materials and methods

### Ethics approval and consent to participate

The study protocol # NAMRU3.PJT.2011.0014 was reviewed and approved by the Naval Medical Research Command Institutional Review Board in compliance with all applicable Federal regulations governing the protection of human subjects (including declaration of Helsinki). The board declared the study as an activity not involving human research and does not require consent to participate (i.e. waiver of consent).

### Surveillance data

This multicenter retrospective study analyzed data from the electronic database of the national Hospital-Acquired Infections (HAIs) surveillance program in Jordan, covering six years from September 2018 to September 2024. Data were obtained from 18 ICUs affiliated with 13 Ministry of Health hospitals across five governorates (Amman, the capital, central part of the Kingdom; Irbid, in the north; Zarqa, in the central region; Salt, in the central region; and Karak, in the south).

Infections detected within 48 h of hospital admission were classified as present on admission, whereas those detected more than 48 h after hospital admission were classified as hospital acquired. Among ICU patients, infections detected more than 48 h after ICU admission were classified as ICU-acquired [16] Four types of HAIs were included in the analysis: bloodstream infections (BSIs), surgical site infections (SSIs), pneumonia, and urinary tract infections (UTIs). Patients with infection episodes with a recovered bacterial isolate and an interpretable carbapenem susceptibility result were included showing carbapenem-resistant (CR) or carbapenem-sensitive (CS) were included in the study. In addition to microbiological data, patient demographics, hospitalization history, length of stay, indwelling medical devices, and clinical outcomes were analyzed.

### Laboratory data

Clinical specimens collected from ICU patients were examined for pathogen identification (ID) and antimicrobial susceptibility testing (AST) at public hospital laboratories according to the concurrent Clinical and Laboratory Standards Institute M100 (CLSI-M100) guidelines [17]. For this study, we included only isolates from each patient’s first HAI episode. Each isolate in the system represented one ICU patient during the study period. Carbapenem-resistant (CR) was identified using either the disk diffusion method or Vitek 2. Results were first reported to the ICUs to guide patient management and subsequently recorded in the electronic surveillance system.

Laboratories enrolled in the national surveillance program are monitored and evaluated by the Central Public Health quality assurance program in Amman.

### Statistical analysis

Data was retrospectively retrieved from the HAIs surveillance system. Categorical variables were summarized as frequencies, and continuous variables as means with standard deviations. Proportion tests compared pathogen distributions between carbapenem-resistant and carbapenem-sensitive groups, with statistical significance set at *P* < 0.05. Mortality odds ratios (ORs) were calculated.

Univariate logistic regression assessed associations between predictor variables and carbapenem-resistant infections, quantified by ORs with 95% confidence intervals (CIs). Significant variables (*P* < 0.05) were included in multivariate logistic regression to identify independent risk factors and control for confounders. ICU categories showing significant associations with resistance were classified into “High-Risk” and “Low-Risk” groups for further analysis.

Predictor variables included patient demographics (gender, age, length of stay), infection type and acquisition (Present on admission, Hospital-acquired, or ICU-acquired), device-associated infections, clinical history, ICU category, and pathogen type. Reference groups for regression analysis were UTI (infection type), present on admission (infection acquisition), Medical Cardiac Care (ICU category), *E. coli* (pathogen type), and non-device-associated infections.

All statistical analyses were conducted using the statistical software R (version 4.2.3).

## Results

### Patient characteristics

Among 1,180 patients, 53% were males, with a median age 55 years (IQR: 3–72 days). A history of prior hospitalization was reported in 52% of cases. Most patients were admitted to the ICU on the day of hospital admission, with a median time of 0 days (IQR: 0–2 days). Medical device use was reported in 88% of ICU patients.

Significant associations were observed for male sex, pneumonia, BSIs, catheter-associated UTI (CAUTI), central line-associated BSIs (CLABSI), ventilator-associated pneumonia (VAP), infections acquired pre-ICU or in the ICU, prior hospitalization, medical device use, and high-risk ICUs (Table 1).

### Pathogen characteristics (Fig. 1)

Between September 2018 and September 2024, 1,976 isolates from ICU-admitted patients in selected public hospitals were reported to the electronic surveillance system. None of the patients reported with HAI were infected with more than one organism.

In total, 65% (1,285/1,976) of the isolates were Gram-negative; with *Klebsiella spp*. (30.8%), *E. coli* (27.6%), *A. baumannii* (20.7%), and *Pseudomonas spp*. (11.4%) being predominant.

### Carbapenem susceptibility testing

Carbapenem-susceptibility testing was performed on 1,180 Gram-negative isolates, revealing 38% (451/1180) as carbapenem-resistant (CR) and 61.8% (729/1180) as carbapenem-sensitive (CS). The CR group was dominated by *A. baumannii* (52%), *Klebsiella spp*. (29%), and *P. aeruginosa* (8%), while the CS group was primarily *E. coli* (41%) and *Klebsiella spp.* (33%) (Fig. 2).By infection type, *A. baumannii* was the leading CR pathogen in BSIs (52.5%), pneumonia (61%), SSIs (69%), and UTIs (39%). Among CS isolates, *Klebsiella spp.* was predominant in BSIs (53%), pneumonia (28%), and UTIs (58%), whereas *E. coli* accounted for most SSIs (41%).Pathogen-specific analysis identified significant associations with *Acinetobacter spp*., *Klebsiella spp*., *Morganella spp*., *Proteus spp*., and *Pseudomonas spp* (Table 2).

### Risk factors

Independent risk factors for CR infections (excluding pathogen type) included: Male sex (OR: 1.22; 95% CI: 1.08–1.86), ICU-acquired infections (OR: 2.5; 95% CI: 1.52–4.12), pneumonia (OR: 8.61; 95% CI: 5.23–14.4), BSIs (OR: 1.91; 95% CI: 1.14–3.23), medical device use (OR: 2.54; 95% CI: 1.13–6.38), high-risk ICU admission (OR: 3.29; 95% CI: 2.26–4.88).

When including pathogen type, significant risk factors remained ICU-acquired infection (OR: 2.44; 95% CI: 1.31–4.54), pneumonia (OR: 2.77; 95% CI: 1.48–5.23), prior hospitalization (OR: 1.38; 95% CI: 0.96–2.0), medical device use (OR: 3.37; 95% CI: 1.28–9.95), and high-risk ICU admission (OR: 3.33; 95% CI: 2.06–5.48). CR infections were significantly associated with *Acinetobacter spp*., *Klebsiella spp*., and *Pseudomonas spp*. Of 1,180 patients, outcomes were recorded for 1003. Mortality in the CR group was 51.3%, with a 3.26-fold higher risk of death compared to the CS group (*P* < 0.001) (Table 3).

## Discussion

In this study, out of the 1,285 Gram-negative pathogens identified, *Klebsiella spp*. was the most common, accounting for 31% of all infections, followed by *E. coli* (28%), *A. baumannii* (21%), and *Pseudomonas spp.* (11%). Several studies have reported similar pathogen distribution patterns across hospital ecosystems, although with varying proportions [5]. Notably, evidence indicates a shift in low- and middle-income countries (LMICs), where Gram-negative bacteria are becoming increasingly prevalent compared to high-income countries (HICs). Factors contributing to this shift include understaffing, limited enforcement of stringent infection prevention and control (IPC) practices, widespread empirical antimicrobial use, and overcrowding in ICUs [18]. Similarly, carbapenem resistance continues to evolve, with significantly higher rates reported in LMICs than in HICs, underscoring global disparities in AMR [1, 19]. In the present study, 38% of Gram-negative isolates were resistant to carbapenem. A comparable proportion (41%) was observed in a similar study by Hummor et al. in Jordan, yet higher than the 26% reported in Turkey [15, 20]. This variation is multifactorial, influenced in part by human factors such as the presence of local or national regulations, antimicrobial stewardship programs, and the availability and accessibility to appropriate drugs [21]. Additionally, behavioral and cultural factors play a role where pharmacists are often seen as key influencers in households, and antibiotics are frequently perceived as a ‘magic pill’ for treating various infections, even for minor ailments like headaches [22]. A key finding of this study that may explain the high resistance levels in ICUs is a history of previous hospitalization, a significant risk factor for acquiring carbapenem resistance. Previous hospital stays often involve exposure to antibiotics, whether prescribed empirically or judiciously, potentially contributing to the development of resistance in these patients [15]. Containment of AMR requires coordinated actions to reduce selective pressure and prevent transmission. Key measures include promoting rational antimicrobial use, strengthening IPC and vaccination programmes, improving surveillance systems, regulating antimicrobial availability and quality, and enhancing collaboration among national and international stakeholders [23].

Among the species most associated with carbapenem resistance, *A. baumannii* was the predominant one (52%). This is a common finding in numerous studies, including studies from Jordan [3, 15, 24, 25]. The prevalence of *A. baumannii* can be attributed to its well-known multidrug-resistant nature. Additionally, this pathogen can persist for extended periods in hospital environments and on inanimate surfaces and is easily transmitted via the hands of healthcare workers, allowing it to exploit the compromised immune systems of ICU patients. This persistence and resistance severely limit treatment options for these vulnerable patients [26, 27]. Logistic regression analysis has identified several risk factors associated with acquiring carbapenem resistance: infection acquisition plays an undeniable role in the development of carbapenem resistance. Hospital settings and in particular ICUs, were significantly associated with developing carbapenem resistance in patients. This finding has been frequently reported in other studies, as ICUs provide a favorable environment for the proliferation of resistant bacteria due to prolonged antibiotic use, patients’ compromised immune status, and extended exposure to a hospital ecosystem rich in resistant microorganisms [28, 29]. The type of infection is also instrumental in acquiring carbapenem resistance. The present study observed a significant association of pneumonia and BSIs with carbapenem resistance, given that *A. baumanii* is the predominant pathogen in this study, its high propensity for acquiring carbapenem resistance is expected. *A.baumanii* has been confirmed to be the source of a broad range of infections in addition to colonization of mechanical ventilators and tracheal secretions owing to its wide dissemination in the hospital environment [30]. Since this pathogen can inhabit the water supply of many hospitals, simple infection control practices, such as avoiding flushing nasogastric tubes with tap water and keeping clean ventilator circuits separate from contaminated ones, can significantly impact the infection rates [31]. Additionally, given the particularities of the Middle East region, geopolitical conflicts have resulted in large-scale displacement of immigrants and refugees, with limited access to appropriate health services, including proper diagnosis and therapeutic options, which further contributed to the spread of multidrug-resistant pathogens, enhanced resistance patterns, and rising incidence rates [32]. The time of admission to the first culture and infection confirmation was significantly longer in the CR group than in the CS group, indicating a prolonged length of stay (LOS) before CR infection diagnosis. This finding is consistent with previous studies identifying ≥ 7 days threshold as a risk factor for acquiring Carbapenem resistance [33, 34]. However, the LOS was not significantly different between the two groups, suggesting that post-infection ICU was not a major contributing factor. This may be due to the higher mortality rate in the CR group, which reduced overall hospital stay [31]. Medical devices were also identified as risk factors in this study, a finding consistent with Han’s study [28], which attributed the rise in infections to an “increased point of entry of pathogens and thus increasing the resistance level”.

Patients with CR infections are more likely to undergo complications and eventually succumb to the disease. The mortality rate of the present study was 51%, a rate consistent with the average fatality rate of other studies [34, 35]. This finding can be attributed to the associated comorbidities and the complications from severe infections with limited treatment options.

High mortality rates can also be attributed to *A.baumanii*, the most prevalent ICU pathogen, which is associated with high hospital mortality [36, 15].

### Limitations

The retrospective nature of our study, limited clinical data, and comorbid conditions pose significant challenges in assessing risk factors and outcomes that can be applied to the general population. Additionally, comparisons among facilities in different governorates should be interpreted cautiously because of the significant differences in institutional policies in infection prevention and control and disease management. For this study, only isolates tested against carbapenems were included. The discrepancy between the total number of isolates and those tested was due to the limited availability of carbapenem discs or the antimicrobial drug in hospital pharmacies during the study period.

## Conclusion and recommendations

These findings underscore the need for strategies to prevent and control carbapenem-resistant infections, particularly in high-risk populations such as patients with invasive devices and those diagnosed with pneumonia. Enhanced infection control measures, including strict hand hygiene practices, judicious antibiotic stewardship, and proper management of invasive devices, are essential to minimize the transmission of carbapenem-resistant bacteria in healthcare settings.

**Fig. 1:**
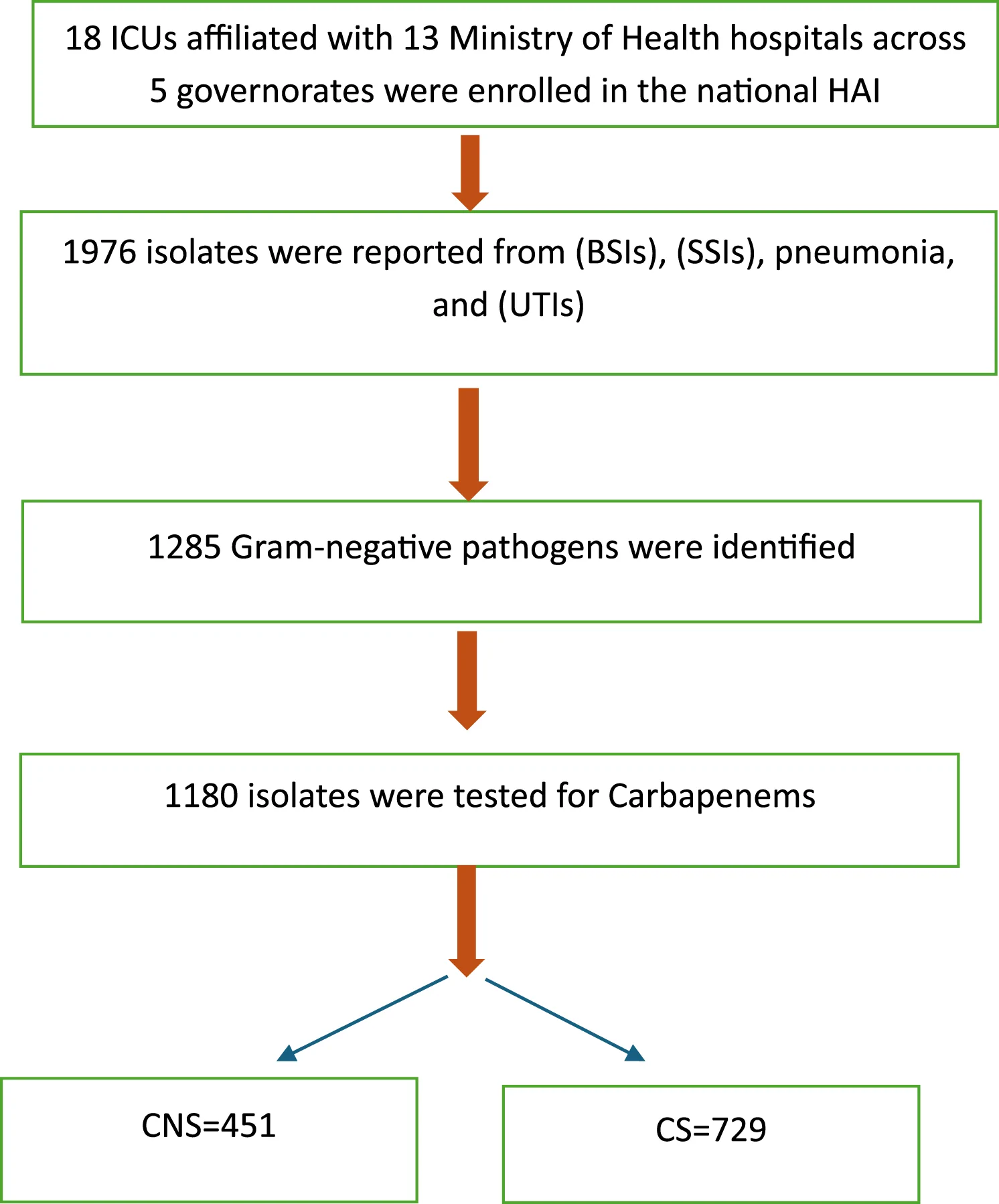
Flow diagram of patient and isolate selection from the national HAI surveillance database, Jordan, (September 2018 – September 2024)

**Fig. 2:**
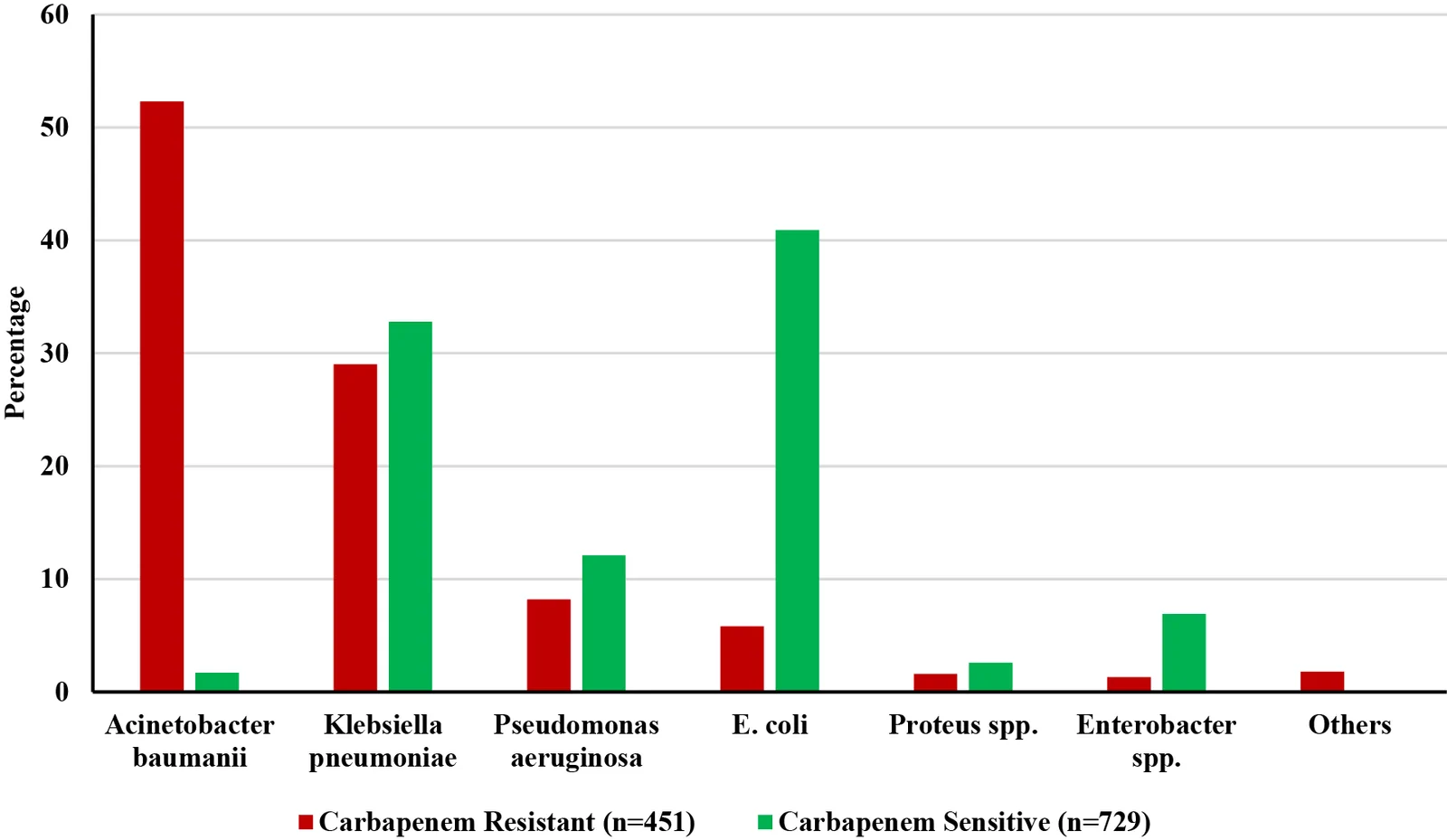
Pathogen distribution based on carbapenem susceptibility (*n* = 1180)

**Table 1:** Comparison of demographic and clinical characteristics between carbapenem-sensitive (CS) and Carbapenem-Resistant (CR) Gram-Negative Bacteria (GNB) groups

Characteristic	Total (*n* = 1180)	CS GNB(*n* = 729)		CR GNB(*n* = 451)		*P* value
		*n*	%	*n*	%	
**Sex**						
Female	553	361	65.28	192	34.72	
Male	627	368	58.69	259	41.31	
**Age**						
Median (IQR1-IQR-3)	55 (IQR 3–72)	51 (IQR 0–71)		61 (IQR 36–73)		< 0.001
**LOS**						
Median (IQR1-IQR3) (Before pathogen isolation)	8 (IQR 1–9)	4(IQR 1–13)		14 (IQR 7–28)		< 0.001
Median (IQR1-IQR3) (After pathogen isolation)	9 (IQR 4–19)	8 (IQR 4–18)		9 (IQR 3–22)		0.15
**Infection Type**						
UTI	535	379	70.84	156	29.16	< 0.001
Pneumonia	186	58	31.18	128	68.82	< 0.001
BSI	387	246	63.57	141	36.43	0.037
SSI	72	46	63.89	26	36.11	0.040
**Device Associated**						
Not Device Associated	734	510	69.48	224	30.52	< 0.001
CAUTI	257	149	57.98	108	42.02	< 0.001
CLABSI	138	57	41.30	81	58.70	< 0.001
VAP	51	13	25.49	38	74.51	< 0.001
**Infection acquisition**						
Present on hospital admission	455	337	74.07	118	25.93	< 0.001
Hospital-acquired	98	55	56.12	43	43.88	0.229
ICU-acquired	627	337	53.75	290	46.25	0.045
**Previous Hospitalization**	618	351	56.80	267	43.20	< 0.001
**Patient Had Surgical Operation**	227	135	59.47	92	40.53	0.037
**CR Vs CS**						
***Acinetobacter spp.***	248	CR = 236	(95.2%)	CS = 12	(4.8%)	X^2^=415.188 *p* < 0.001
***Klebsiella spp.***	343	CR = 127	(37.0%)	CS = 216	(63.0%)	X^2^ =1.045 *p* = 0.307
***Pseudomonas spp.***	122	CR = 37	(30.3%)	CS = 85	(69.7%)	X^2^=4.591 *p* = 0.032
**Mortality**	507	263	(69.8%)	244	(42.3%)	*p* < 0.001
Mortality OR (95% CI)			3.149 (2.392–4.145)			

LOS; length of stay, UTI, urinary tract infection, BSI; bloodstream infection, SSI, surgical site infection; CAUTI; catheter associated urinary tract infection, CLABSI, Central line associated blood stream infection, VAP, ventilator associated pneumonia, ICU, intensive care unit, *P* < 0.05 is significant

**Table 2:** Univariate logistic regression analysis of risk factors for carbapenem-resistant gram-negative bacterial infections

Characteristic	OR	95% CI	*p*-value
**Patient Sex**	1.32	1.05, 1.68	< 0.001
**UTI**	**Reference Group**		
Pneumonia	5.36	3.75, 7.74	< 0.001
BSI	1.39	1.05, 1.84	0.019
SSI	1.37	0.81, 2.28	0.228
**Not Device Associated**	**Reference Group**		
CAUTI	1.65	1.23, 2.21	< 0.001
CLABSI	3.24	2.23, 4.72	< 0.001
VAP	6.66	3.57, 13.2	< 0.001
**Present on hospital admission**	**Reference Group**		
Hospital-acquired	2.23	1.42, 3.50	< 0.001
ICU-acquired	2.46	1.90, 3.20	< 0.001
**Previous Hospitalization**	1.44	1.13, 1.83	0.003
**Patient Had Surgical Operation**	1.07	0.79, 1.43	0.667
***E. coli***	**Reference Group**		
*Klebsiella spp.*	6.63	4.26, 10.7	< 0.001
*Acinetobacter spp.*	222	144, 470	< 0.001
*Pseudomonas spp*	4.91	2.83, 8.64	< 0.001
Others	2.78	1.48, 5.19	< 0.001
**Patient Had Device**	7.39	3.76, 16.7	< 0.001
**Medical Cardiac Care**	**Reference Group**		
Burn Critical Care	4.27	0.48, 33.0	0.159
Medical/Surgical Critical Care	4.64	1.90, 13.9	< 0.002
Medical Critical Care	9.53	3.89, 28.7	< 0.001
Medical Neurological Care	3.3	1.15, 11.0	< 0.001
Neonatal ICU	1.99	0.79, 6.08	0.175
Pediatric Medical Critical care	1.08	0.36, 3.68	0.890
Surgical Cardiothoracic Critical care	7.47	2.56, 25.5	< 0.001
Surgical Critical Care	4.62	1.86, 14.0	< 0.002
Trauma Critical Care	3.82	1.47, 11.9	< 0.010

UTI: urinary tract infection, BSI: bloodstream infection, SSI: surgical site infection; CAUTI; catheter associated urinary tract infection, CLABSI: Central line associated blood stream infection, VAP: ventilator associated pneumonia, ICU: intensive care unit, NICU: Neonatal intensive care unit, *P* < 0.05 is significant

**Table 3:** Multivariate logistic regression analysis of risk factors for carbapenem-resistant gram-negative bacterial infections

Characteristic	OR	95% CI	*p*-value
**Patient Sex**	1.22	0.87, 1.71	0.256
**Infection Acquisition**			
Present on hospital admission	—	—	
Hospital-acquired	1.83	0.89, 3.73	0.099
ICU-acquired	2.44	1.31, 4.54	0.004
**Infection Type**			
UTI	—	—	
Pneumonia	2.77	1.48, 5.23	0.001
BSI	0.61	0.31, 1.17	0.139
SSI	0.47	0.16, 1.34	0.163
	—	—	
**Device Associated**			
Not Device Associated	—	—	
CAUTI	0.71	0.35, 1.44	0.342
CLABSI	1.56	0.79, 3.06	0.198
VAP	0.45	0.16, 1.26	0.126
**Previous Hospitalization**	1.38	0.96, 2.00	0.084
**Patient Had Device**	3.37	1.28, 9.95	0.019
**History of Surgical Operation**	0.7	0.43, 1.13	0.151
**ICU Risk Groups**			
Low Risk ICUs	—	—	
High Risk ICUs	3.33	2.06, 5.48	< 0.001
**Pathogens**			
* E. coli*	—	—	
* Klebsiella spp.*	5.83	3.59, 9.74	< 0.001
* Pseudomonas spp.*	4.52	2.49, 8.30	< 0.001
* Acinetobacter spp.*	220	105, 506	< 0.001
Other	2.5	1.27, 4.89	0.008

UTI: urinary tract infection, BSI: bloodstream infection, SSI: surgical site infection; CAUTI; catheter associated urinary tract infection, CLABSI:Central line associated blood stream infection, VAP: ventilator associated pneumonia, ICU: intensive care unit, NICU: Neonatal intensive care unit, P< 0.05 is significant

## Data Availability

The datasets used and/or analysed during the current study are available from the corresponding author on reasonable request.

## Declarations

### Disclaimer

M.S. is an employee of the U.S. Government. This work was prepared as part of her official duties. Title 17, U.S.C. § 105 provides that copyright protection under this title is not available for any work of the U.S. Government. Title 17, U.S.C. § 101 defines a U.S. Government work as work prepared by a military service member or employee of the U.S. Government as part of that person’s official duties.

## Abbreviations

CR-GNB: Carbapenem-resistant Gram-negative bacteria
ICUs: Intensive Care Units
WHO: World Health Organization
LMICs: low and Middle-Income Countries
HAIs: Hospital-Acquired Infections
BSIs: Bloodstream Infections
SSIs: Surgival Site Infections
UTIs: Urinary Tract Infections
CR: Carbapenem Resistace
CS: Carbapenenm Sensitive
ID: Identification
AST: Antimicrobial Susceptibility Testing
MIC: Minimum Inhibitory Concentration
Ors: Odds Ratios
Cis: Confidence Intervals
CAUTI: Catheter-associated Urinary Tract Infection
CLABSI: Central Line-associated Blood Stream infection
VAP: Ventilator-associated Pneumonia
LOS: Length of Stay
